# The rare hemoglobin variant Hb Mizuho: report of a Swiss family and literature review

**DOI:** 10.1007/s00277-021-04458-3

**Published:** 2021-02-15

**Authors:** Linet Njue, Cesare Medri, Peter Keller, Miriam Diepold, Behrouz Mansouri Taleghani, Alicia Rovó

**Affiliations:** 1 Department of Hematology and Central Hematology Laboratory, Inselspital, Bern University Hospital, University of Bern, 3010 Bern, Switzerland; 2 Hematology, Spital Langenthal SRO AG, 4900 Langenthal, Switzerland; 3 Hematology and Oncology, Children’s Hospital, Inselspital, Bern University Hospital, University of Bern, 3010 Bern, Switzerland

**Keywords:** Hemoglobinopathy, Hemoglobin, Rare, Erythrocytapheresis

## Abstract

Hb Mizuho is a very rare unstable hemoglobin; here, we describe the clinical history of three Swiss family members with Hb Mizuho together with a systematic review of the previously six published cases. The clinical history of the adult woman we report here is unique since this is the first Hb Mizuho presenting with Moyamoya complications and the first case reported with long-term erythrocyte exchange. The literature review showed that Hb Mizuho was mainly reported as a de novo mutation, with the exception of children descended from known cases. All published patients with this unstable hemoglobin showed severe hemolytic anemia with the exception of one; all were regularly transfused. Patients with higher HbF levels might require fewer transfusions. All patients underwent splenectomy at a median age of 4 years and had variable clinical improvement; some achieved complete resolution of transfusion dependency after splenectomy. Iron overload in Hb Mizuho patients seems to be mainly attributed to transfusions and has less to do with ineffective erythropoiesis. Diagnosis might be challenging; a normal hemoglobin electrophoresis should not rule out the diagnosis of unstable hemoglobin in patients with otherwise unexplained hemolytic anemia. This series shows the enormous utility of using molecular techniques for diagnosis.

## Introduction

Hemoglobin (Hb) Mizuho is a very rare hemoglobinopathy associated with congenital chronic hemolysis. Only six cases have been reported worldwide to date. A heterozygous T→C mutation at position 68 of the β-chain with a Leu→Pro substitution causes a severely unstable hemoglobin and is clinically characterized by hemolytic anemia often leading to transfusion dependency. Here, we report Hb Mizuho in three members of a Swiss family, a currently 41-year-old Swiss woman and her two children.

## Case description

### Case 1

The patient was born full term in 1979, after an uncomplicated pregnancy and delivery with a birth weight of 2.88 kg. Her hemoglobin levels dropped quickly to a level of 75 g/l within the first 2 months of life. Fetal hemoglobin (HbF) and adult hemoglobin A2 (HbA_2_) levels were at this point 1.8% and 2.9% respectively. The clinical presentation was interpreted as severe coombs negative hemolytic anemia of unknown etiology. Isopropanol test and erythrocyte enzymes were normal. Regular transfusion therapy was initiated at the age of 2 months, and she was henceforth transfusion-dependent with an average requirement of two transfusions per month during her first 3 years of life. She underwent splenectomy at the age of 3, which contrary to the expected effect did not halt transfusion dependency. Iron chelation therapy was initiated at age 15 with ferritin levels in the range of 7000 μg /l and has been continued with few interruptions up to date. Transfusion-related alloimmunization has never occurred.

**Fig. 1:**
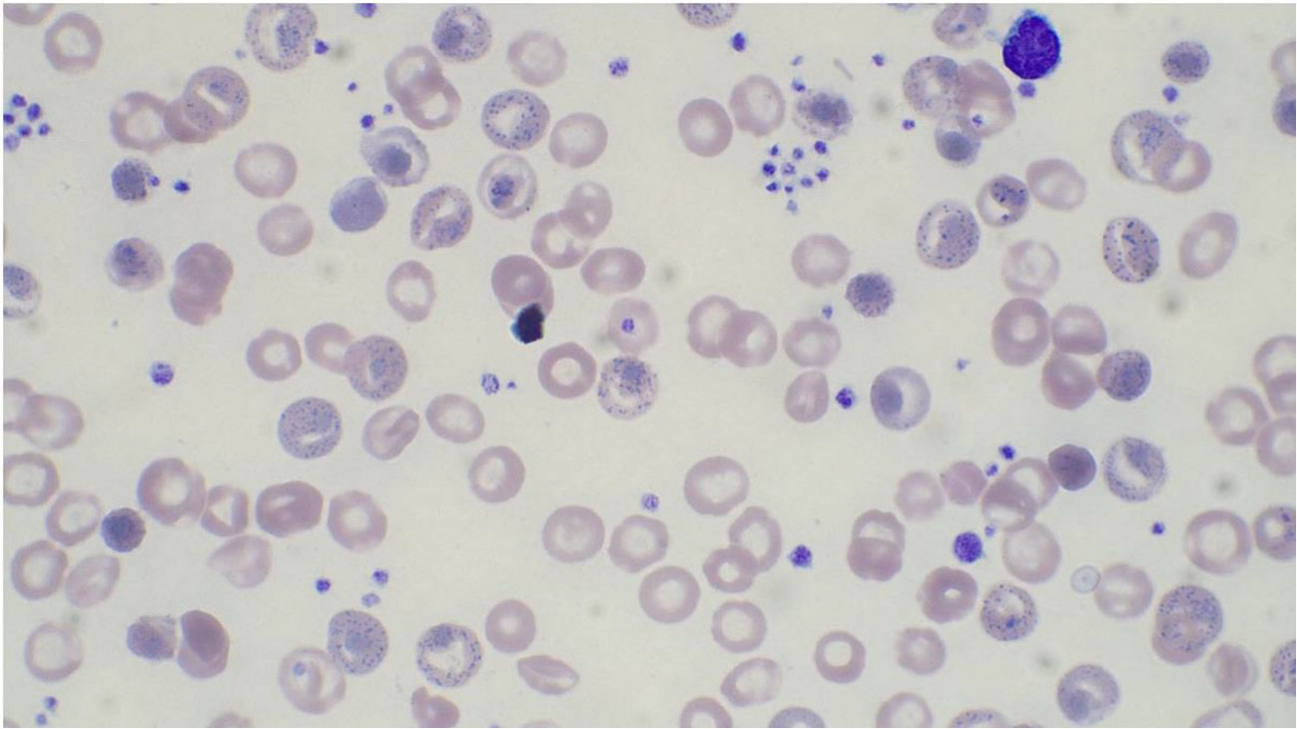
Peripheral blood smear of case 1 using Giemsa staining. Remarkable signs of dyserythropoiesis with basophilic stippling. This blood smear was 7 weeks after the last red cell exchange apheresis

In the peripheral blood smear, remarkable basophilic stippling, Heinz bodies, and reticulocytosis were evident (Fig. 1). A bone marrow examination initially carried out at the age of 2 revealed hypercellularity for age with prominent erythroid proliferation. A bone marrow examination repeated later at the age of 22, mainly due to persistent neutrophilia and lymphocytosis, showed similar findings. Conventional karyotyping was normal.

When the patient was 23 years old, a new investigation for her hemolytic anemia was initiated. Her initial hemoglobin electrophoresis had yielded normal results. Blood samples had to be delivered to a different hospital, meaning that denaturing of the unstable hemoglobin may have occurred before testing. The presence of an unstable hemoglobin was first detected when the test was performed immediately after blood withdrawal, directly in the laboratory. An anomalous hemoglobin was identified running before HbA_2_. Subsequent DNA analysis of the beta-globin gene revealed a mutation in one of the two alleles at codon 68 leading to a change from leucine (CTC) to proline (CCC).

At the age of 25, therapy with hydroxyurea at a dose of 2000 mg daily was initiated and administered for a total of 33 months; her Hb F levels however did not increase (remained in the range of 2 to 3%), and the therapy was therefore discontinued. Transfusions of two red cell concentrates were still required monthly.

**Fig. 2:**
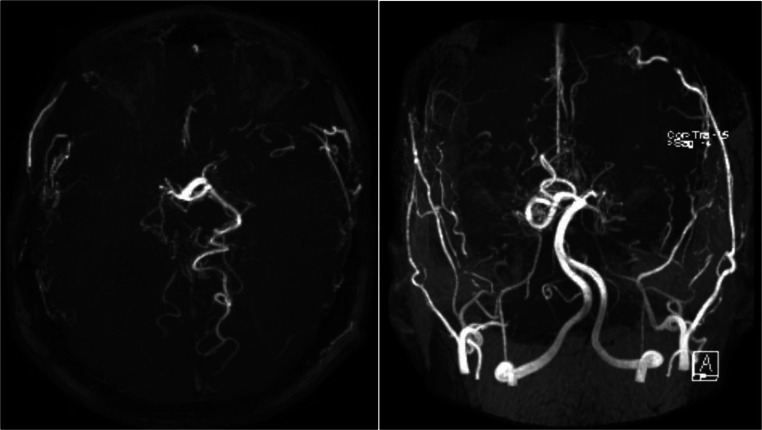
MRI angiography of case 1 showing bilateral internal carotid artery stenosis leading to Moyamoya diagnosis. This was prior to surgical revascularization

At the age of 25, the patient presented with aphasia and right-sided hemiparesis. Moyamoya, a disease characterized by occlusion of cerebral vessels with development of collateral vessels, in this case bilateral internal carotid artery stenosis, was diagnosed (Fig. 2). A surgical bypass revascularization was performed without complications, and following surgery, 100 mg aspirin was prescribed and is currently ongoing.

Since the most probable cause for the cerebrovascular complication was the underlying hemoglobinopathy, a re-assessment of the therapy was warranted. Red blood cell exchange apheresis every 6 weeks was therefore initiated at the age of 25 and has been ongoing and generally well tolerated up to date. Cholecystectomy was performed due to symptomatic cholelithiasis at the age of 26.

Her first pregnancy at the age of 24 was uneventful without significant aggravation of hemolysis. Regular transfusions were continued throughout the pregnancy, while iron chelation was put on hold. During her second pregnancy at the age of 30, red blood exchange apheresis was continued monthly.

The patient was able to complete her primary and secondary education and thereafter trained as a professional chef. She currently receives partial disability pension due to the complications of her disease and works part time. She describes good quality of life without relevant restrictions in her daily activities.

Her two children were diagnosed with Hb Mizuho in early infancy. Her eldest child is now 17 years old and currently does not require regular transfusions. Her now 10-year-old son requires transfusions every 6 to 12 weeks.

### Case 2: her daughter

**Fig. 3:**
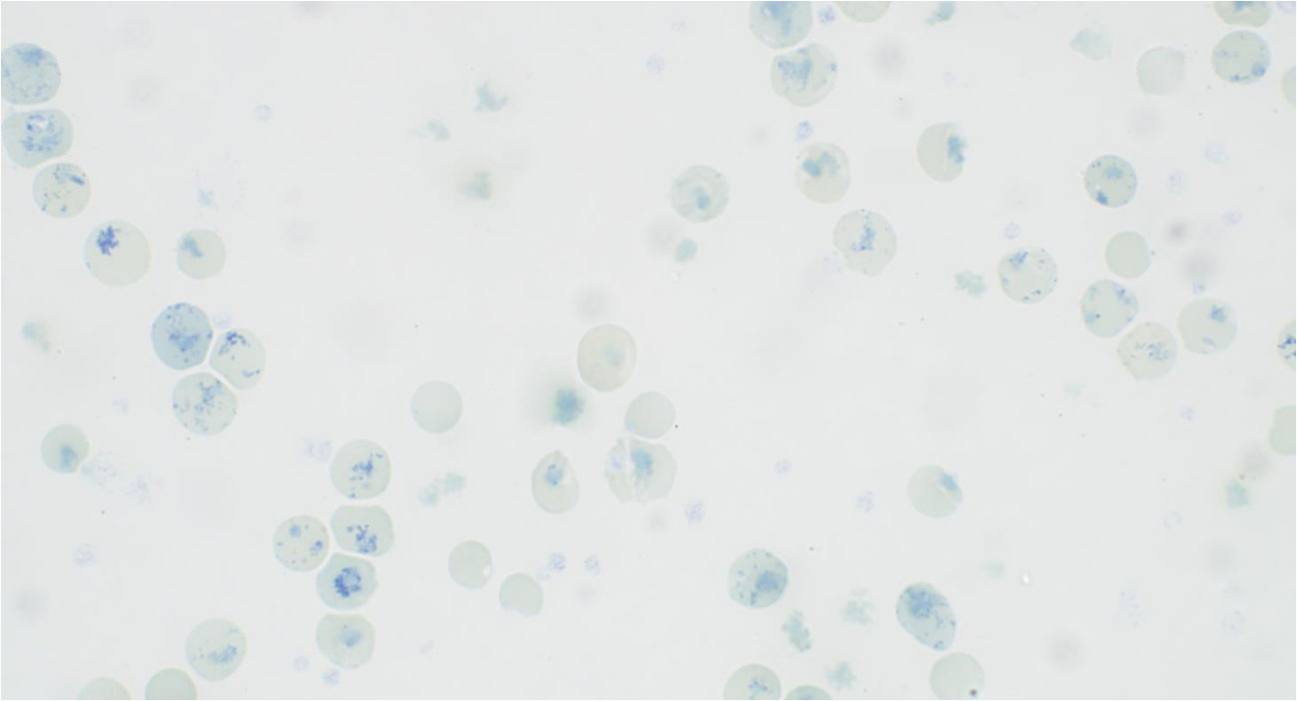
Peripheral blood smear of case 2. Methylene blue staining showing reticulocytosis and some faintly visible Heinz bodies

This is a 17-year-old female patient. She was born by C-section due to breech positioning at a birth weight of 2.2 kg and Apgar score of 8/9/9. Her hemoglobin level 2 days after birth was 204 g/l. This decreased significantly to 57 g/l with 274 G/l reticulocytosis (13%) at the age of 7 months. Regular transfusion therapy every 2 to 7 weeks was subsequently initiated. HbF level at the age of 4 years was 1.4%. DNA sequence analysis of the beta-globin gene revealed the same mutation as in her mother. Iron chelation therapy with deferasirox was begun at the age of 5 having ferritin levels of 3400 μg/l. Despite elective splenectomy at the age of 7, transfusions every 8 weeks were still required. During follow-up, it was possible to prolong the transfusion intervals from monthly to roughly once every 8 months. Cholecystectomy was performed due to symptomatic cholelithiasis at the age of 10. At the age of 12 as her transfusion dependency decreased, deferasirox was discontinued. Her current hemoglobin levels are in the range of 80–85 g/l with reticulocytes of 1200–2000 G/l (85%) (Fig. 3), and her ferritin values remain in the normal range of 60–70 μg/l. She also depicts high lactate dehydrogenase (LDH) levels of 1200–1400 U/L and total bilirubin levels in the range of 60 μmol/l as well as an undetectable haptoglobin. She is an active teenager, interested in sports and dance, and describes a normal quality of life.

### Case 3: her son

This is a 10-year-old boy who currently requires transfusions every 6 to 12 weeks. Due to preterm labor at 37 weeks, he was born via C-section with a birth weight of 2.3 kg and Apgar score of 9/9/9. Regular transfusions every 4 to 6 weeks via port catheter were first initiated at the age of 4 months, after his hemoglobin levels dropped below 75 g/l. At 3 years of age, ferritin levels reached 1552 μg/L; iron chelation therapy with deferasirox was begun at a dose of 250 mg/d (= 18 mg/kg); this has been well tolerated up to date. His current ferritin values have consistently been below 500 μg/l, and therefore deferasirox has been discontinued. In an effort to decrease transfusion dependency and iron overload, a partial splenectomy was performed at the age of 6. A noticeable decrease of transfusion requirement from once every 6 to 12 weeks has been observed during the course of time; the transfusion threshold was set at a hemoglobin value of 80 g/l. He is currently doing well in 3rd grade at primary school.

Both children have repeatedly been screened for Moyamoya disease by magnetic resonance angiography with negative results.

Due to the scarce information available on this unusual hemoglobinopathy, we aimed to review all the cases in order to delineate the most relevant characteristics of the published cases to date (Table 1).

**Table 1:** Characteristics of patients with hemoglobin Mizuho—a systematic review.

Author	*N*	Sex	Country of origin	Year of birth	Hb F %/age at determination	Transfusion dependency	Transfusions frequency	Sx/timeline	Need of Transfusion post-Sx	Clinical follow-up	Bone marrow aspirate
Ohba et al. 1977	1	F	Japan	1971	6.8%/3 years	Yes	Monthly, start 18 months	Yes, at 4 years	No	NA	Erythroid hyperplasia
Labotka et al. 1990	2	F	Italy	1986	NA	Yes	Monthly, start 6 months	Yes, at 3 years	No	NA	Hypercellular erythroid hyperplasia
Keeling et al. 1991	3	M	America	1986	35%/2 years 22%/4 years	No	Unknown	Yes, at 4 years	No	NA	NA
Harthoorn et al. 1994	4	M	The Netherlands	1989	31%/16 months 16%/3 years 9.8%/5 years	Yes	Every 8 weeks, start 2 years	Yes, at 6 years	No	Papillary thyroid carcinoma, Gilbert’s syndrome; hepatic failure leading to liver transplant	Active erythropoiesis
Perera et al. 2015 Family (2 members)	5	F	Sri Lanka	1969	NA	Yes	NA	Yes, early age	Yes	NA	NA
	6	M	Sri Lanka	1996	NA	Yes	NA	Yes, early age	Yes	NA	NA
Njue et al. Family (3 members)	7	F	Switzerland	1979	1.8%/2 years	Yes	Approx. monthly, start at 2 months	Yes, at 3 years	Yes, RBC exchange every 6 weeks	Moyamoya disease	Erythroid hyperplasia
	8	F	Switzerland	2003	1.4%/4 years	Yes	Monthly, start 7 months	Yes, at 7 years	Every 6–10 months	None relevant	Not performed
	9	M	Switzerland	2009	N/A	Yes	Monthly, start 4 months	Yes, at 6 years	Every 3 months	None relevant	not performed

## Discussion

Hb Mizuho is very rare. To our knowledge, only 6 cases have been reported in the literature [1–6], with only one adult patient. Hb Mizuho was first reported in a Japanese girl (1), next in an Italian girl (2), and later in boys from USA (3) and the Netherlands (4). Hb Mizuho has recently been identified in two family members from Sri Lanka (5). Hb Mizuho was mainly reported as a de novo mutation, with the exception of the two children reported here and the Sri Lankan boy.

Here, we describe the courses of three Swiss family members with Hb Mizuho with transfusion dependency since early infancy due to macrocytic, normochromic anemia with severe chronic hemolysis. Interestingly, splenectomy did not lead to resolution of transfusion dependency in any of them. Removal of the spleen ideally prolongs red blood cell survival by reducing red blood cell sequestration and may ultimately result in the reduced need for blood transfusions. Splenectomy has been shown to reduce transfusion requirements in patients with thalassemia [7, 8] as well as sickle cell disease [9]. In unstable hemoglobins, splenectomy should be considered when there is severe anemia and/or massive or symptomatic splenomegaly [10]. In the mother, there was practically no change in transfusion requirement post-splenectomy; the children however have profited mildly.

Variants of unstable hemoglobin represent a diagnostic challenge in rare cases of congenital hemolytic anemia. The lack of previous family history can guide the diagnosis in the wrong direction and delay the diagnosis. Easier access to molecular tests has greatly facilitated diagnosis in recent years. The first of our three cases clearly illustrates this difficulty prior to easy access to molecular diagnosis. Despite the fact that hemolysis was detected right after birth, several rounds of diagnostic work-up remained unsuccessful with a confirmed diagnosis only at the age of 23 years. This was only possible with analysis of fresh blood, sampled directly in the laboratory.

The clinical history of case 1 is unique since this is the first Hb Mizuho presenting with Moyamoya complications and the first case reported with long-term erythrocyte exchange.

Moyamoya disease is a very rare disorder characterized by progressive stenosis and occlusion of the internal carotid arteries and arteries of the circle of Willis and its major branches. Suzuki and Takaku introduced the term “Moyamoya disease” to describe the numerous collateral vessels at the base, which resembled a “puff of smoke” [11–13]. A single etiologic agent has not been identified, although toxic, infectious, and inflammatory etiologies have been proposed, but never proven [14]. Moyamoya disease has previously been associated with sickle cell disease [15]. Moyamoya has also been associated with other hemolytic anemias including hemoglobin E-beta thalassemia as well as the rare hemoglobin Fairfax with beta-thalassemia and Southampton hemoglobinopathy [16–19]. Putting all this into consideration, a correlation between Moyamoya and Hb Mizuho in our case is, though not proven, likely.

Furthermore, patients with chronic hemolytic anemia are known to be at a higher risk of thrombotic complications suggestive of a hypercoagulable state, the mechanism of coagulation activation likely being of multifactorial origin [20]. Regarding the association of Hb Mizuho and thrombosis, we have not found other cases with thrombosis reported in the literature. It must be emphasized that the patient presented here developed Moyamoya disease at the age of 25. Since elevated free plasma hemoglobin caused by hemolysis is toxic for the vascular endothelium, this is a probable cause for the vasoocclusive events in our patient.

This case suggests that patients with Hb Mizuho could be at risk of thromboembolic complications and thrombosis prophylaxis may be warranted in particular cases.

Our index patient has profited from red blood cell exchange apheresis. Recurrent vasoocclusive events have since not occurred, and her iron values are stable. Sufficiently repressing the production of the harmful, aberrant hemoglobin could therefore be considered beneficial as secondary prophylaxis after a thromboembolic event. In red cell exchange, red blood cells (RBCs) containing abnormal Hb are removed and replaced by healthy volunteer donor RBCs. Red cell exchange can be performed by manual exchange or by automated exchange using a blood cell separator (erythrocytapheresis). Compared to simple RBC transfusions, this procedure offers several advantages, such as lower risk for iron accumulation and efficient control of pathological erythrocyte populations. The most frequent indication for RBC exchange is sickle cell disease; this is the standard treatment in patients with a history of or a risk for acute stroke and is a clinical option for other acute complications of sickle cell disease [21].

In this case, the treatment with hydroxyurea did not bring any clinical benefit; further conclusions are no possible due to the lack of data in the literature reporting experiences using hydroxyurea in this hemoglobinopathy.

Iron overload is of clinical relevance in many hemoglobinopathies. Iron overload develops from increased intestinal iron absorption signaled by hepcidin suppression secondary to ineffective erythropoiesis [22], while it can also be secondary to regular transfusions. The degree of toxicity may also vary depending on the disease.

Iron overload in Hb Mizuho patients seems to be mainly attributed to transfusions and has less to do with ineffective erythropoiesis. The degree of iron overload seems to present itself with different patterns. The type and dosing of chelation therapy should be adapted according to the individual’s course. Both oral and intravenous iron chelators are effective.

Due to the scarce information available on this unusual hemoglobinopathy, we aimed to review all the cases in order to delineate the most relevant characteristics of the published cases to date. Thus, the patients described in the literature had severe hemolytic anemia and with the exception of one; all were regularly transfused (Table 1). Remarkable basophilic stippling, Heinz bodies, and reticulocytosis seem to be a common feature in all these cases. All patients underwent splenectomy at a median age of 4 years (range 3–7) and had variable clinical improvement after splenectomy. One patient did reasonably well until the age of 4 years (3) and was not transfusion dependent even before splenectomy. Splenectomy in this case, which was performed due gradually worsening anemia (hemoglobin 72 g/l at the age of 4 years) and marked splenomegaly (spleen size 349 gr), improved his condition considerably. Three patients achieved complete resolution of transfusion dependency after splenectomy (cases 1, 2, and 4), supporting the benefit of this procedure.

Hemoglobin F level seems to be helpful in the follow-up of Hb Mizuho. A reported case with mild clinical presentation had a large quantity of HbF up to the age of 5 years (HbF levels of 35% and 22% at the ages of 2 and 4 respectively) (3). The Dutch boy described by Harthoorn et.al. (4) similarly had relatively high HbF levels, which could explain why transfusion dependency began much later at the age of 2 years. In the two of our patients, where HbF was measured, levels were normal, nicely explaining transfusion dependency in these patients.

Information regarding the clinical courses of Hb Mizuho patients through to adulthood is scarce. This forth reported patient from the Netherlands was diagnosed with papillary thyroid carcinoma, for which he was curatively treated with a total thyroidectomy plus lymph node dissection and radioactive iodine therapy at the age of 13 years. He later developed fulminant hepatic failure requiring liver transplantation at the age of 25 years. Interestingly, Gilbert syndrome was also diagnosed. The authors describe a possible connection between the hemoglobinopathy and the liver disease, as it is known in intrahepatic cholestasis of sickle cell disease [6].

A normal hemoglobin electrophoresis should not rule out the diagnosis of unstable hemoglobin in patients with otherwise unexplained hemolytic anemia, since unstable hemoglobin variants that undergo rapid denaturation or are represented in the HbA fraction might not be detected by this method [23]. This could lead to under- or misdiagnosis. Though repeat testing should be encouraged in unclear cases, this series shows the enormous utility of using molecular techniques for diagnosis. The numerous tests initially performed in our reported cases, including isopropanol stability tests, erythrocyte fragility tests, heat denaturation test, high performance liquid chromatography, and hemoglobin electrophoresis, did not yield conclusive results. Examination of hemoglobin stability by heat or isopropanol is a useful screening test in detection of unstable hemoglobins. Stability testing is performed by incubating with isopropanol, whereby unstable hemoglobins yield visible precipitates. Similarly, in heat stability testing, hemolysate is incubated for 1–2 h at 50 °C. Unstable hemoglobins yield a visible precipitate, while normal hemoglobins do not [24, 25]. Correcting pre-analytic errors in our index patient however led to the diagnosis. With this exception, identification of the variant required sequencing of a segment of amplified DNA that included the beta-globin gene. Bone marrow examinations did not contribute to the diagnosis either. The only relevant finding in the five cases where bone marrow examination was performed was erythroid hyperplasia.

It must be highlighted that the first Hb Mizuho case was reported in 1977 with the following 3 cases reported in the early 1990s. The algorithm for hemoglobinopathy testing has since evolved enormously [23]. Red cell indices and morphology followed by separation and measurement of Hb fractions using protein-based methods (Hb electrophoresis and high performance liquid chromatography (HPLC)) are nowadays the methods of choice for initial qualitative and quantitative Hb analyses. Hb variants can usually be detected using these methods. A survey done by van Zwieten et al. [26] involving 20,000 blood samples showed that high performance liquid chromatography analysis followed by globin gene sequencing of rare variants is an effective method to reveal Hb variants. DNA-based methods are used as the following step for variants with ambiguous or unusual results from protein analysis. Cappellini et al. [27] illustrate the recommended diagnostic flowchart for identification of thalassemia, which can be applied for suspected Hb variants.

Molecular diagnostic possibilities have grown enormously in the past decades. This paper aims to raise awareness that rarer and not necessarily inherited diseases can nowadays easily be diagnosed.
